# Ultraviolet vision in birds: the importance of transparent eye media

**DOI:** 10.1098/rspb.2013.2209

**Published:** 2014-01-07

**Authors:** Olle Lind, Mindaugas Mitkus, Peter Olsson, Almut Kelber

**Affiliations:** Department of Biology, Lund University, Lund, Sweden

**Keywords:** ocular media transmittance, ultraviolet sensitivity, colour vision, bird, evolution

## Abstract

Ultraviolet (UV)-sensitive visual pigments are widespread in the animal kingdom but many animals, for example primates, block UV light from reaching their retina by pigmented lenses. Birds have UV-sensitive (UVS) visual pigments with sensitivity maxima around 360–373 nm (UVS) or 402–426 nm (violet-sensitive, VS). We describe how these pigments are matched by the ocular media transmittance in 38 bird species. Birds with UVS pigments have ocular media that transmit more UV light (wavelength of 50% transmittance, *λ*_T0.5_, 323 nm) than birds with VS pigments (*λ*_T0.5_, 358 nm). Yet, visual models predict that colour discrimination in bright light is mostly dependent on the visual pigment (UVS or VS) and little on the ocular media. We hypothesize that the precise spectral tuning of the ocular media is mostly relevant for detecting weak UV signals, e.g. in dim hollow-nests of passerines and parrots. The correlation between eye size and UV transparency of the ocular media suggests little or no lens pigmentation. Therefore, only small birds gain the full advantage from shifting pigment sensitivity from VS to UVS. On the other hand, some birds with VS pigments have unexpectedly low UV transmission of the ocular media, probably because of UV blocking lens pigmentation.

## 1. Introduction

To be able to see ultraviolet (UV) light, an eye has to meet two criteria: it has to possess UV-sensitive (UVS) photoreceptors and ocular media that transmit this part of the spectrum. Human eyes meet the first criterion but not the second: one of our pigments has an absorbance maximum (*λ*_max_) at 430 nm and is sensitive to UV light, but our lens contains carotenoids and acts as a long-pass cut-off filter that transmits less than 5% of the incident light at wavelengths shorter than 400 nm [1].

Although the UV transmittance of the eye media determines our own insensitivity to short wavelength light, almost all studies on UV vision in animals focus on visual pigments. The eye media—cornea, aqueous humour, lens and vitreous humour—have evolved for being transparent but even in the absence of pigments, radiation of wavelengths below 310 nm is strongly absorbed by components, such as nucleic acids and aromatic amino acids [2]. Few species have been investigated for ocular media transmittance (OMT) and comparative studies are only available for fishes [3–5] and jumping spiders [6]. How is OMT related to UV vision in other animals with lens eyes?

**Figure 1.**
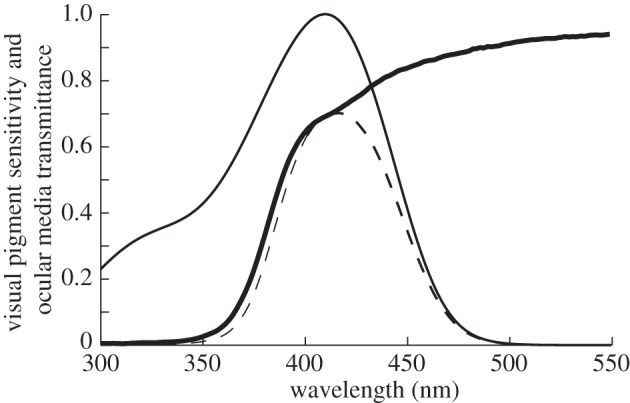
The effect of OMT on VS-cone sensitivity. Solid thin line: the sensitivity of an unscreened sws1-pigment expressed in a VS-cone (*λ*_max_ 405 nm); solid thick line: OMT of the common swift (table 1); dashed line: sws1-pigment screened by the ocular media. Ocular media screening decreases absolute sensitivity, shifts the absorbance peak to longer wavelengths and limits the visual range in the UV.

Birds are of special interest because their UVS sws1-pigments come in two variants, the most common violet-sensitive (VS) pigment with peak sensitivity (*λ*_max_) between 402 and 426 nm, and the UVS pigment in parrots and some passerines with peak sensitivity between 360 and 373 nm [7]. Single point mutations result in a shift from VS to UVS pigments, and bird UVS pigments have arisen several times independently [8], whereas the three other bird visual pigments are remarkably conserved [9]. The sensitivity of bird UVS/VS cones is set by the combination of OMT and the sensitivity of the UVS/VS pigment ([10]; figure 1). How well does OMT fit visual pigment absorbance?

There seems to be a trend for birds with UVS pigments to have ocular media transmitting light of shorter wavelengths compared with birds with VS pigment [11]. Ödeen & Håstad [8] assume that only birds with highly UV-transparent ocular media have an advantage of UVS pigment and expect to find birds with UV-transmitting eye media and VS pigments, but no birds with UV-blocking eye media and UVS pigments. Is this true and how does OMT influence colour discrimination ability and sensitivity of bird eyes?

Finally, Hart [7] hypothesized that smaller birds are more likely to have UVS pigments than larger birds. Is OMT—and thus UV-sensitivity—in birds set by eye size? In this study, we address these three issues by investigating OMT in 38 species from 11 orders of bird and discuss our findings in the context of visual sensitivity and evolution.

## 2. Material and methods

### (a) Data collection

We measured OMT of eyes from birds that had to be euthanized because of injuries or health conditions in a bird rescue programme in southern Sweden. Eyes from Timor zebra finches (*Taeniopygia guttata*) and Bourke's parrots (*Neopsephotus bourkii*) were acquired from animals euthanized in other research projects, eyes from burrowing owls were obtained from Copenhagen Zoo. Published OMT data on 10 bird species were kindly provided by Nathan Hart at The University of Western Australia (table 1; N. S. Hart 2012, personal communication). Published OMT data from ostrich (*Struthio camelus*), mallard (*Anas platyrhynchos*), bowerbirds and green-backed firecrown (*Sephanoides sephaniodes*) were acquired by scanning publications with Plot Digitizer [27], remaining OMT data are from earlier publications by various authors (table 1).

**Table 1.** Species included in the study. (The sws1-pigments are classified by type and when known, their *λ*_max_ is given in nanometres. *n*_OMT_ gives the number of individuals and (number of eyes) for which OMT was averaged. A dash indicates missing data.)

order	common name	species name	sws1 pigment	reference pigment	*λ*_T0.5_ (nm)	*n*_OMT_	reference OMT
Psittaciformes	Bourke's parrot	*Neopsephotus bourkii*	UVS	[8,12,13]^b^	334	3(3)	this study
	budgerigar	*Melopsittacus undulatus*	UVS, 371	[14]	320	3(5)	[10]
	crimson rosella	*Platycercus elegans*	UVS, 363	[12]	319	1(2)	[12]
Passeriformes	common starling	*Sturnus vulgaris*	UVS, 362	[15]	337	5^c^	[15]
	common blue tit	*Cyanistes* (*Parus*) *caeruleus*	UVS, 372	[16]	316	1(1)	[16]
	great tit	*Parus major*	UVS	[8,13,17]^b^	314	1(2)	this study
	common blackbird	*Turdus merula*	UVS, 373	[16]	343	1(1)	[16]
	song thrush	*Turdus philomelos*	UVS	[8,13,17]^b^	335	1(1)	this study
	Gouldian finch	*Erythrura gouldiae*	UVS, 370	[18]	315	1(1)	[18]
	cut-throat finch	*Amadina fasciata*	UVS, 370	[18]	316	1(1)	[18]
	white-headed munia	*Lonchura maja*	UVS, 373	[18]	317	1(1)	[18]
	plum-headed finch	*Neochmia modesta*	UVS, 373	[18]	314	1(1)	[18]
	Timor zebra finch	*Taeniopygia guttata*	UVS, 359	[14]	321	2(4)	this study
	rook	*Corvus frugilegus*	VS	[13]^b^	365	2(4)	this study
	common magpie	*Pica pica*	VS	[13]^b^	370	2(3)	this study
	green catbird	*Ailuroedus crassirostris*	VS, 406	[19]	340	1(1)	[19]
	great bowerbird	*Chlamydera nuchalis*	VS, 404	[19]	349	1(1)	[19]
	regent bowerbird	*Sericulus chrysocephalus*	VS, 408	[19]	349	1(1)	[19]
	satin bowerbird	*Ptilonorhynchus violaceus*	VS, 410	[19]	344	1(1)	[19]
	spotted bowerbird	*Chlamydera maculata*	VS	[19]	351	1(1)	[19]
Strigiformes	burrowing owl	*Athene cunicularia*	VS	^a^	359	1(2)	this study
	northern long-eared owl	*Asio otus*	VS	^a^	356	1(1)	this study
	tawny owl	*Strix aluco*	VS	^a^	353	1(2)	this study
	boreal (Tengmalm's) owl	*Aegolius funereus*	VS	^a^	335	1(1)	this study
Falconiformes	Eurasian buzzard	*Buteo buteo*	VS, 405	[13]	375	1(2)	[20]
	Eurasian sparrowhawk	*Accipiter nisus*	VS, 405	[13]	369	1(2)	[20]
	red kite	*Milvus milvus*	VS	[13]^b^	394	1(2)	[20]
	common kestrel	*Falco tinnunculus*	VS	[13]^b^	379	1(2)	[20]
Struthioniformes	ostrich	*Struthio camelus*	VS, 405	[21]	369	1(1)	[21]
Galliformes	domestic chicken	*Gallus gallus domesticus*	VS, 418	[14]	351	2(4)	[10]
	wild turkey	*Meleagris gallopavo*	VS, 420	[22]	355	1(1)	[22]
	Indian peafowl	*Pavo cristatus*	VS, 421	[23]	364	1(2)	[23]
Apodiformes	common swift	*Apus apus*	VS	[24]^b^	388	1(2)	this study
	green-backed firecrown	*Sephanoides sephaniodes*	VS	[24]^b^	310	2(—)	[11]
Columbiformes	rock dove	*Columba livia*	VS, 404	[14]	337	4(7)	[10]
Procellariiformes	wedge-tailed shearwater	*Puffinus pacificus*	VS, 406	[25]	335	1(1)	[25]
Podicipediformes	great crested grebe	*Podiceps cristatus*	VS	[8]	390	1(2)	this study
Anseriformes	mallard	*Anas platyrhynchos*	VS, 420	[26]	371	—	[26]

^a^Owls are grouped as birds with VS-pigment although this is uncertain, see main text for details.

^b^The type of sws1-pigment is inferred from phylogeny.

^c^OMT is based upon an average transmittance from five lenses, one aqueous humour, four corneas and one vitreous humour.

### (b) Measurement of ocular media transmittance

We measured OMT in a dark room, following the same protocol as in our recent study on raptors [20]. We started measurements within 1 h after the point of death and completed them within 2 h. We enucleated the eye, removed the sclera, choroid and retina from the posterior pole of the eye with a circular cut, half the transverse diameter of the eye, leaving the vitreous humour intact. We rinsed the eye with 340 mosmol kg^−1^ phosphate buffered saline (PBS)-solution and placed it with the posterior pole up in a custom-made matte black plastic container with a circular (5 mm) fused silica window in the bottom. Depending on eye size, we used one of four containers (diameters and heights of 33, 30; 21, 19 mm; 17, 14; 12, 10 mm), stabilized the eyes using metal washers and filled the container with PBS-solution. Light from a PX2-Xenon lamp (Ocean Optics) illuminated the cornea via a light guide through the fused silica window and transmitted light was collected by a second light guide connected to a spectroradiometer (Maya, Ocean Optics) controlled by Spectrasuit software (v. 1.0, Ocean Optics). We used different light guides (200, 600 and 1000 μm, all with a numerical aperture of 0.22, Ocean Optics) and aligned light guides with the container in a microbench system (LINOS). The transmittance of the container with washers and PBS-solution was measured as reference. The sampling base was 1 nm, three to five measurements were averaged for each eye, smoothed by an 11-point running average and normalized to the highest value within the range 300–700 nm. From these data, we determined the wavelength at which 50% of the light incident on the cornea was transmitted to the retina, *λ*_T0.5_, a commonly used indicator of UV transparency [7], and the slope of the OMT function. See the electronic supplementary material for more details.

### (c) Eye size

As a measure of eye size, we used the axial length (distance from corneal vertex to posterior sclera) of the eye, either measured with a calliper in freshly excised eyes prior to measurements of the OMT, or in hemisected frozen eyes following the protocol of Lind & Kelber [28]. Freezing has a negligible effect on path length in this context [23]. Data for 10 species were obtained from the literature, for the great tit (*Parus major*), we estimated axial length by using the axial length of yellowhammer (*Emberiza citrinella*) that has similar size and same eye diameter [29,30].

### (d) Modelling colour vision

Using the receptor-noise limited model [31] (see the electronic supplementary material for a summary) and D65 spectrum [32] as illumination source, we calculated how colour discrimination thresholds for UV colours were affected by OMT and sws1-pigment type. We considered reflectance spectra with a distinct Gaussian peak in the UV region and a second peak at longer wavelength. The initial spectrum was inspired by reflectance spectrum of the green breast of the budgerigar (*Melopsittacus undulatus*) [33] with a narrow peak (36 nm full width at half-maximum) at 320 nm and broader peak at 520 nm.

We determined colour discrimination thresholds by decreasing the amplitude of the UV peak in steps of 1% until colour contrast between the initial and the reduced UV peak reached above 1 just noticeable difference. We noted this threshold value for colours with UV peaks at each nanometre between 320 and 420 nm (with corresponding shifts of the long wavelength peak) and repeated the calculations for colours with a broad UV peak (96 nm full width at half-maximum).

We performed calculations for four visual systems, combining VS pigment and UVS pigment with either high or low UV transmittance of the ocular media. As model species, we chose the domestic chicken (*Gallus gallus domesticus*) for VS pigments and the budgerigar for UVS pigments (see the electronic supplementary material, table S1 for details).

## 3. Results

### (a) Variation in ocular media transmittance among birds

**Figure 2.**
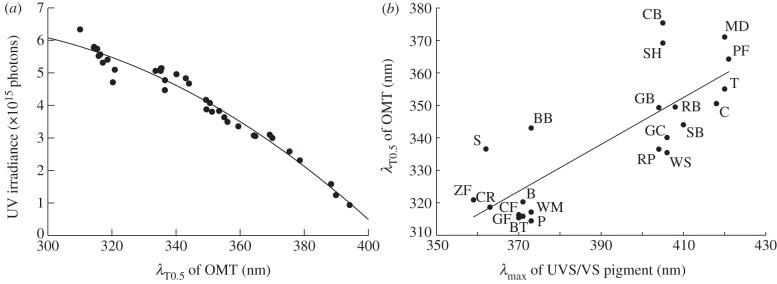
The correlation between OMT (*λ*_T0.5_), retinal UV irradiance and peak position of UVS/VS pigment (*λ*_max_). (*a*) UV irradiance decreases with increasing *λ*_T0.5_ of the OMT (Spearman's rank correlation, *n* = 36, *p* < 0.001). The line indicates the best fit by a second-order polynomial function. UV irradiance is determined as the number of photons of 300–400 nm reaching the retina, assuming a D65 daylight spectrum [32]. Each filled circle represents one species; all species (table 1) were included in analysis, except ostrich and mallard, for which OMT is unknown at very short wavelengths. (*b*) Correlation between OMT (*λ*_T0.5_) and the spectral tuning of pigments (*λ*_max_) is significant (Spearman's rank correlation, *n* = 22, *p* < 0.001) and best described by a linear function. B, budgerigar; BB, common blackbird; BT, common blue tit; C, domestic chicken; CB, Eurasian buzzard; CF, cut-throat finch; CR, crimson rosella; GB, great bowerbird; GC, green catbird; GF, Gouldian finch; MD, mallard; P, plum-headed finch; PF, Indian peafowl; RB, regent bowerbird; RP, rock pigeon; S, common starling; SB, satin bowerbird; SH, Eurasian sparrowhawk; T, wild turkey; ZF, Timor zebra finch; WM, white-headed munia; WS, wedge-tailed shearwater.

Among all 38 species included in this study, *λ*_T0.5_ varied between 310 and 394 nm (table 1), and the amount of UV light reaching the retina was negatively correlated with *λ*_T0.5_ of OMT (figure 2*a*). Passerines that include species with UVS pigments and species with VS pigments were the order with the largest variation, with *λ*_T0.5_ ranging from 314 to 370 nm.

For the subset of 22 species with known pigment spectra (table 1), *λ*_T0.5_ of OMT correlated with *λ*_max_ of the sws1-pigment (figure 2*b*). While *λ*_T0.5_ of OMT are distributed rather evenly, pigment sensitivities cluster, with UVS pigments having *λ*_max_ between 359 and 373 nm and VS pigments having *λ*_max_ between 404 and 421 nm. Within birds with the same pigment type (UVS or VS), *λ*_T0.5_ of OMT is not correlated with pigment sensitivity (Spearman's rank correlations; UVS, *n* = 10, *p* = 0.43; VS, *n* = 12, *p* = 0.29).

**Figure 3.**
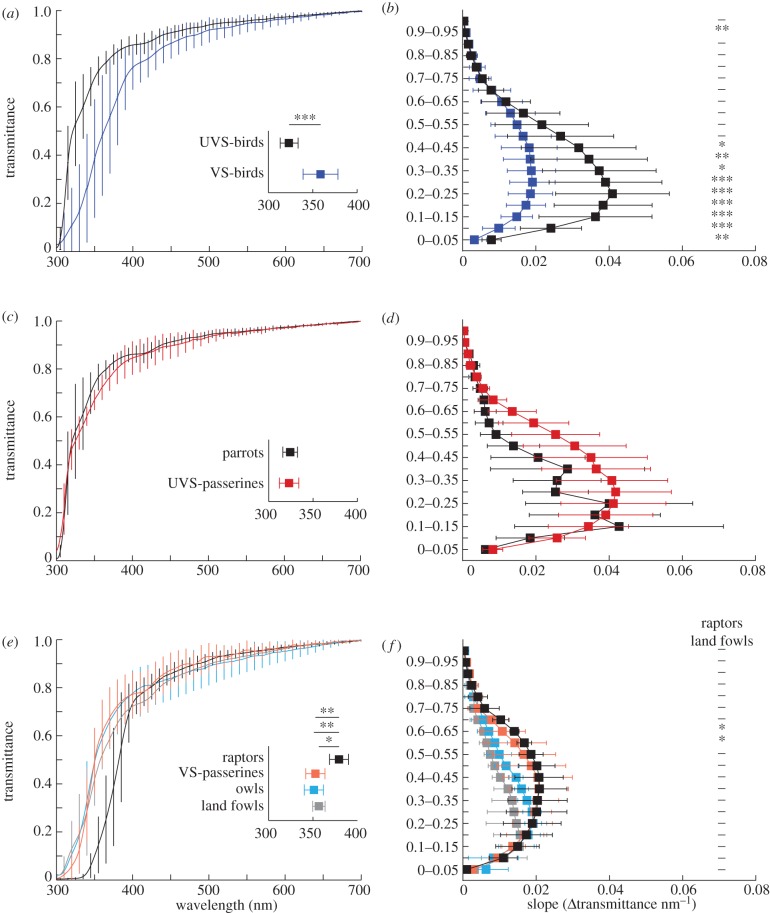
The variation in OMT between bird groups. Average OMT for; (*a*) birds with UVS and VS pigment; (*c*) parrots and UVS-passerines; (*e*) raptors, owls, land fowls and VS-passerines. Insets show the average wavelength position of 50% transmittance (*λ*_T0.5_) for each group. In (*e*), significant differences in *λ*_T0.5_ are for (top down) raptor and VS-passerines, raptors and owls, raptors and land fowls. The slopes of the OMT functions in (*a*,*c*,*e*) are shown as average difference in transmittance per nanometre for each 5% transmittance interval in (*b*,*d*,*f*). Error bars indicate ±1 s.d. and significance levels are: *p < 0.05, **p < 0.01, ***p < 0.001 (two-tailed unpaired *t*-test for normally distributed data and otherwise two-tailed Wilcoxon rank sum test). The significance levels in (*b*,*d*,*f*) were corrected for multiple comparisons (*n* = 20) using the Dunn-Šidák method [35]. A dash (or the absence of notation) indicates no significance. All transmittance spectra are available in the electronic supplementary material.

We therefore compared OMT (*λ*_T0.5_ and slope of the OMT function) between all 13 birds known to have UVS pigments and all 25 birds with VS pigments. For owls, no sws1-pigment has been found so far [9,34] but it remains to be seen whether it is truly lost. Here, we group owls among birds with VS pigments, but our conclusions do not change if owls are excluded. On average, birds with UVS pigments have OMT functions with lower *λ*_T0.5_ (323.2 ± 10.0 nm; mean ± s.d.) than species with VS pigments (358.4 ± 19.6 nm) and the OMT function in the cut-off region is steeper in birds with UVS pigments (figure 3*a*,*b*).

**Figure 4.**
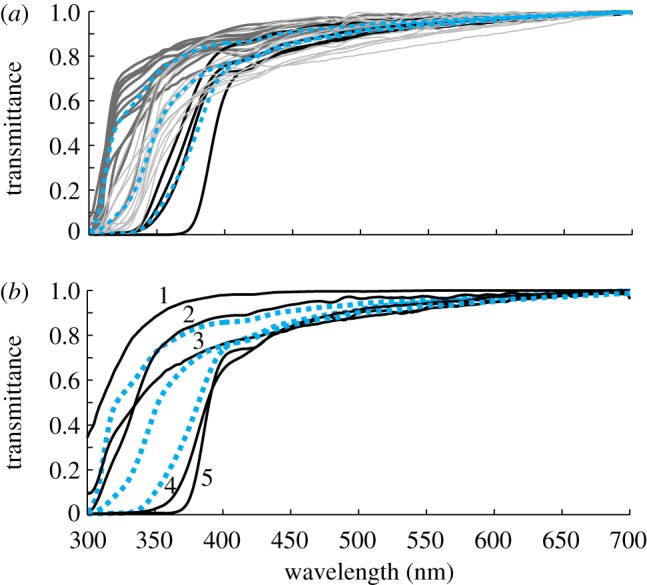
OMT in individual bird species and average, UV-OMT and raptor-OMT (for details see text). (*a*) OMTs of all birds used in the statistical analyses. Thick medium grey lines: UV-OMT (parrots and UVS-passerines); thin light grey lines: V-OMT (owls, land fowls and VS-passerines); black lines: raptors; dashed blue lines: averages for each group. (*b*) Group averages as in (*a*) and OMT functions of additional birds with VS-pigment. (1) Green-backed firecrown; (2) wedge-tailed shearwater; (3) rock pigeon; (4) common swift; (5) great crested grebe.

We then compared bird groups where data from at least three species were available. These are parrots (UVS), land fowls, and owls and raptors (VS). Passerines were divided into species with UVS pigments and species with VS pigments and treated as two groups. We found no significant differences between parrots and UVS-passerines (figure 3*c*,*d*). Among birds with VS pigments, raptors had OMT with higher *λ*_T0.5_ (389.3 ± 10.6 nm) and steeper slopes than land fowls (figure 3*e*,*f*) but we found no differences between owls, land fowls and VS-passerines (351.1 ± 10.8, 356.6 ± 7.0 and 352.7 ± 10.8 nm). The results suggest a grouping of OMTs into three average curves (figure 4*a*): UV-OMT for parrots and UVS-passerines, with high UV transmittance (*λ*_T0.5_ 314–343 nm); V-OMT for owls, land fowls and VS-passerines with medium UV transmittance (*λ*_T0.5_ 344–370 nm); and raptor-OMT with low UV transmittance (*λ*_T0.5_ 369–394 nm).

Among the seven bird species included in this study but not in the statistical tests, some interesting cases are worth mentioning (figure 4*b*). Common swift (*Apus apus*) and great crested grebe (*Podiceps cristatus*) have VS-pigment and ocular media with very low UV transmittance, similar to raptors. By contrast, rock pigeon (*Columba livia*), wedge-tailed shearwater (*Puffinus pacificus*) and green-backed firecrown, also species with VS-pigment, have ocular media with high UV transmission, more similar to birds with UVS pigments.

### (b) Ocular media transmittance and eye size

**Figure 5.**
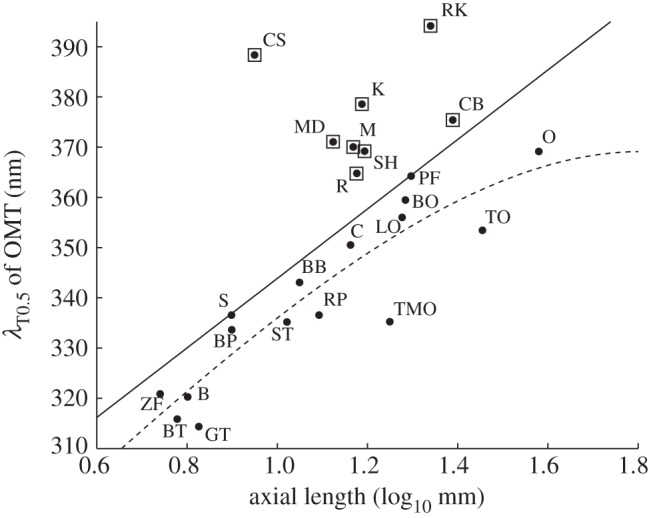
The correlation between eye size (axial length) and OMT (*λ*_T0.5_). Each black dot represents a species. A linear function describes the correlation between axial length and *λ*_T0.5_ best (solid line; Spearman's rank correlation, *p* < 0.001). After excluding some species with low UV transmittance (high *λ*_T0.5_; open squares), the correlation between the variables is best described by a two-term exponential function (dashed line; Spearman's rank correlation, *p* < 0.001). Abbreviations with references to axial length taken from earlier studies: B, budgerigar [28]; BB, common blackbird [29]; BO, burrowing owl; BP, Bourke's parrot [28]; BT, common blue tit; C, domestic chicken; CB, Eurasian buzzard; CS, common swift [36]; GT, great tit (see Material and methods); K, Eurasian kestrel; LO, northern long-eared owl; M, common magpie; MD, mallard [37]; O, ostrich [38]; PF, Indian peafowl [23]; R, rook [39]; RK, red kite; RP, rock pigeon; S, common starling [40]; SH, Eurasian sparrowhawk; ST, song thrush; TMO, boreal (Tengmalm's) owl; TO, tawny owl [41]; ZF, Timor zebra finch.

With the observed peculiarities (figure 4*b*) and the wide range of OMT in passerines in mind (table 1), we tested the hypothesis that transmittance of ocular media for UV light is limited by eye size [7]. For 23 species, for which eye size is known, we found a strong linear correlation between *λ*_T0.5_ of OMT and the axial length of the eyes (figure 5). However, while no bird with large eyes was found to have OMT with very low *λ*_T0.5_, some species with medium-sized eyes had OMT with high *λ*_T0.5_. The exclusion of these species (squares in figure 5) resulted in a two-term exponential function describing OMT of the remaining species as a function of eye size.

### (c) Ocular media transmittance and colour discrimination in the ultraviolet range

To test how OMT and VS/UVS pigments affect colour discrimination thresholds for UV colours, we combined UVS (budgerigar) and VS pigments (domestic chicken) with the average V-OMT of owls, land fowls and VS-passerines, and the average UV-OMT of parrots and UVS-passerines (figure 4).

**Figure 6.**
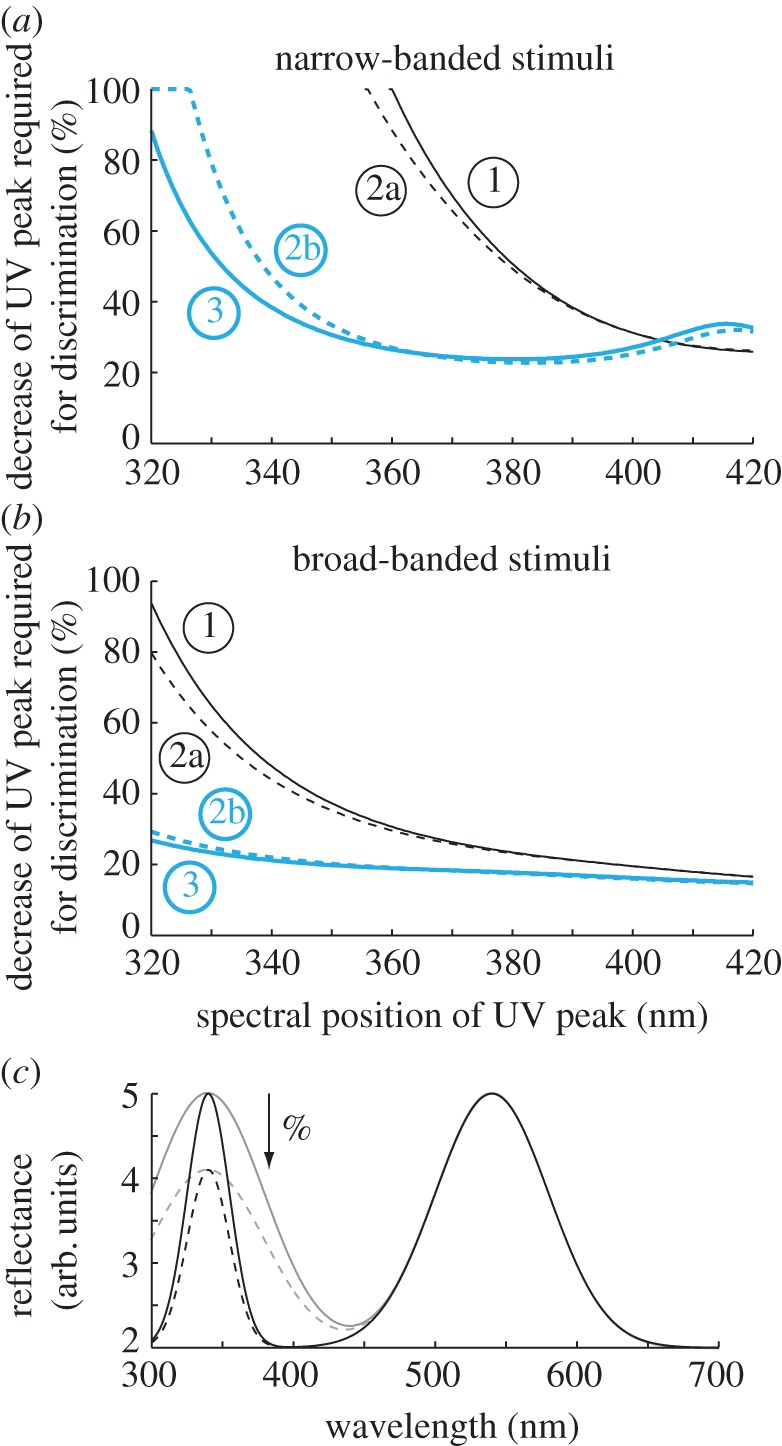
Discrimination thresholds for colours with UV peaks of different amplitude (*a*,*b*) in four visual systems: (1) VS pigment and V-OMT; (2a) VS pigment and UV-OMT; (2b) UVS pigment and V-OMT and (3) UVS pigment and UV-OMT. Thresholds requiring a 100% decrease of the UV peak were considered to represent the limit for no discrimination. (*c*) Examples of one narrow-banded (solid black line) and one broad-banded (solid grey line) stimulus with UV peaks at 340 nm illustrated with hypothetical amplitude differences at threshold (dashed black and dashed grey lines, respectively). For further explanations, see text.

For narrow-band UV stimuli, figure 6*a* shows that a bird with a VS pigment and V-OMT (figure 6*a*, line 1) was incapable of discriminating any amplitude difference at wavelengths shorter than 361 nm. A bird with VS pigment but UV-OMT (figure 6*a*, line 2a) could discriminate colours down to 356 nm. If the bird instead had UVS pigments, but V-OMT (figure 6*a*, line 2b), discrimination was possible down to 327 nm. Discrimination of differences at 320 nm was only possible with UVS pigments and UV-OMT (figure 6*a*, line 3). Interestingly, the bird with VS pigment and V-OMT had a slight advantage at longer wavelengths and could discriminate smaller differences in peak amplitude of a stimulus at 420 nm than UVS birds. We found similar but less pronounced results with broad-peaked stimuli (figure 6*b*) and with plumage spectra from parrots and passerines (see the electronic supplementary material).

## 4. Discussion

### (a) Visual pigments and the variation of ocular media transmittance among birds

Our results from 38 species of birds confirm earlier observations [7,11] that birds with UVS pigments have ocular media that transmit UV radiation of shorter wavelengths than birds with VS pigments (figures 2*b*–4). However, within each group, we find no correlation between pigment tuning and OMT (using 22 species with measured pigment *λ*_max_ data). We propose separate average curves; UV-OMT for parrots and passerines with UVS pigments, V-OMT for passerines with VS pigments, owls, and land fowls, and raptor-OMT (figure 4).

For modelling colour discrimination in bright light, these results are promising. For species with unknown OMT, it seems acceptable to adopt one of the three average OMT functions outlined in figure 4*a*, or to use the OMT of a closely related species. There is however, a need to be cautious, because not all birds follow the general patterns (figure 4*b*). For instance, two apodiform species have extremely different OMT (table 1 and figure 4*b*), for unknown reasons. Data on shearwater, mallard and grebe (table 1), and a study on gull OMT [42] indicate high variability within water birds, which we plan to investigate further. The habitat of water birds, especially of diving species, may require very specific adaptations of ocular media.

### (b) Eye size and the variation of ocular media transmittance in lens eyes

We found a strong correlation between OMT (*λ*_T0.5_) and eye size (figure 5). This is expected for unpigmented ocular media, where OMT is determined by UV absorption by amino acids and scattering [2,7,43], as has been shown in some groups of fishes [4,5]. Some species of jumping spiders also seem to have unpigmented ocular media, although the correlation between OMT and eye size is unclear [6]. Our data (figure 5) are, to our knowledge, the first to support the hypothesis that bird sensitivity to short UV wavelengths is constrained by the size of the eye [7].

In both fishes and jumping spiders, species with unpigmented ocular media and species with clearly pigmented ocular media and lower UV transmittance have been found [3,6,44,45]. Our data suggest that some birds (e.g. raptors and swifts in which UV transmittance is not correlated with eye size) probably also have pigmented ocular media. We hope to find these lens pigments in a subsequent project. It remains to be shown how small-eyed birds avoid retinal damage by UV radiation [46].

### (c) Raptors and other birds with low ultraviolet transmittance

Diurnal raptors may not be a monophyletic group [47] but all species included in this study have ocular media with exceptionally low UV transmittance (figures 3 and 4; [20]). This is probably an example of convergent adaptation and we see two possible reasons for blocking UV light. First, raptors have the highest visual acuity of all animals [48] and suffer the most from the strong lens aberrations at short wavelengths. Specifically, chromatic aberration may deteriorate the image for the short wavelength sensitive cone too much if short wavelength light is transmitted. Second, the damaging effect of UV radiation on the retina [46] may be strong in birds hunting on the wing in the open. These reasons could be relevant for swifts as well, another example of a bird hunting moving prey on the wing, with similarly low transmission of UV light to the retina (figures 4*b* and 5).

### (d) Ocular media transmittance and colour vision—can we understand evolution?

As earlier [10], we found that the transmittance of the ocular media affects colour discrimination in bright light to a small degree (figure 6; electronic supplementary material). However, it has been shown that many birds favour strong UV reflectance of the plumage of potential mates [49] and low discrimination thresholds may be very important. In bright light, a bird with UVS pigment and highly UV transparent ocular media clearly performs better than a bird with VS pigment and less UV transparent ocular media. The latter cannot discriminate intensity variations at wavelengths below 361 nm. Still, the contribution of OMT to this difference is small especially if we exclude raptors and other birds that probably have pigmented lenses. Only for the discrimination of stimuli at very short wavelengths, for example the green-UV breast of budgerigars, are both UVS pigments and UV-OMT required.

For the detection of weak UV signals or colour discrimination in dim light, the situation may be different. The absorbance by the ocular media greatly reduces the absolute sensitivity of the UVS/VS cones (figure 1; [10,42]) and absolute sensitivity becomes important when photons are scarce. For instance, hollow-nesting birds, and birds active in dim light, may need high UV transmittance of ocular media to detect differences in UV reflections from conspecifics, eggs or nestlings.

Our modelling results do not fully disclose the evolutionary sequence of events leading to the transition from VS birds to UVS birds. Contrary to the expectation [42], a bird with low OMT for UV light can benefit from evolving a UVS pigment (figure 6). However, there are few examples of this; nearly all birds with known UVS pigments have UV-OMT (table 1). The reason for this may be that a bird cannot use the full advantage of the pigment transition, for example detection of weak short wavelength UV signals, without an accompanying shift in the OMT. In addition, there are likely costs and benefits associated to the transitions yet unknown and many bird species, for instance the large cassowaries with putative UVS pigment [8], remain to be investigated.

Interestingly, if highly UV transparent ocular media truly were required for the evolutionary transitions from VS to UVS pigments to be stable, our correlation between OMT and eye size suggests that this evolutionary path was open to already small bird species only. It seems less likely that the evolutionary sequence could be the reverse, with a VS to UVS pigment transition occurring in large birds first, then followed by a quick reduction in body and eye size that makes the transition beneficial and stable.

## Notes

All of the methods used in this study are in accordance with the policy and law of the Swedish Board of Agriculture and the Swedish Environmental Protection Agency.
